# Characterization and Duodenal Transcriptome Analysis of Chinese Beef Cattle With Divergent Feed Efficiency Using RNA-Seq

**DOI:** 10.3389/fgene.2021.741878

**Published:** 2021-10-05

**Authors:** Chaoyun Yang, Liyun Han, Peng Li, Yanling Ding, Yun Zhu, Zengwen Huang, Xingang Dan, Yuangang Shi, Xiaolong Kang

**Affiliations:** ^1^ School of Agriculture, Ningxia University, Yinchuan, China; ^2^ Ningxia Agriculture Reclamation Helanshan Diary Co.Ltd., Yinchuan, China

**Keywords:** residual feed intake, feed efficiency, weighted gene co-expression network analysis, beef cattle, duodenal

## Abstract

Residual feed intake (RFI) is an important measure of feed efficiency for agricultural animals. Factors associated with cattle RFI include physiology, dietary factors, and the environment. However, a precise genetic mechanism underlying cattle RFI variations in duodenal tissue is currently unavailable. The present study aimed to identify the key genes and functional pathways contributing to variance in cattle RFI phenotypes using RNA sequencing (RNA-seq). Six bulls with extremely high or low RFIs were selected for detecting differentially expressed genes (DEGs) by RNA-seq, followed by conducting GO, KEGG enrichment, protein-protein interaction (PPI), and co-expression network (WGCNA, *n* = 10) analysis. A total of 380 differentially expressed genes was obtained from high and low RFI groups, including genes related to energy metabolism (ALDOA, HADHB, INPPL1), mitochondrial function (NDUFS1, RFN4, CUL1), and feed intake behavior (CCK). Two key sub-networks and 26 key genes were detected using GO analysis of DEGs and PPI analysis, such as TPM1 and TPM2, which are involved in mitochondrial pathways and protein synthesis. Through WGCNA, a gene network was built, and genes were sorted into 27 modules, among which the blue (*r* = 0.72, *p* = 0.03) and salmon modules (*r* = −0.87, *p* = 0.002) were most closely related with RFI. DEGs and genes from the main sub-networks and closely related modules were largely involved in metabolism; oxidative phosphorylation; glucagon, ribosome, and N-glycan biosynthesis, and the MAPK and PI3K-Akt signaling pathways. Through WGCNA, five key genes, including FN1 and TPM2, associated with the biological regulation of oxidative processes and skeletal muscle development were identified. Taken together, our data suggest that the duodenum has specific biological functions in regulating feed intake. Our findings provide broad-scale perspectives for identifying potential pathways and key genes involved in the regulation of feed efficiency in beef cattle.

## Introduction

In the beef industry, feed provision accounts for more than 70% of total input costs (Patience et al., 2015). Reducing feed expenditure has gradually become a significant focus on farm animal research. Residual feed intake (RFI) — defined as the difference between the average daily feed intake (ADFI) and the expected daily feed intake (EDFI) required for maintenance and food production (meat, eggs, milk, etc.) (Koch et al., 1963)— is an indicator of feed efficiency in an animal. A low or negative RFI value indicates greater-than-average utilization efficiency (Yan et al., 2017). In contrast, a high or positive RFI value indicates that the feed efficiency is low and that the animal has faster metabolism and requires frequent feeding and activity (Zhang et al., 2017). As an indicator, RFI is independent of growth traits such as average daily gain (ADG) and body weight (BW) (Baker et al., 2006) and is mainly related to economic traits such as dry matter intake (DMI) and feed conversion efficiency (FCR) (Gomes et al., 2012). Recently, pathways such as adenosine 5′-monophosphate (AMP)-activated protein kinase (AMPK) signaling (Karisa et al., 2014), metabolic pathways and oxidative stress (Tizioto et al., 2017), lipid metabolism (Tizioto et al., 2015), and the immune response (Gondret et al., 2017) were reported to be involved in RFI variance. Moreover, genes such as COL14A1 (de Lima et al., 2020), OGN (Vigors et al., 2019), ACE (Yi et al., 2015), and SMCT (de Lima et al., 2020) and quantitative trait loci such as EFEMP1 (de Lima et al., 2020) and SHC3 (Weber et al., 2016) were also identified to be potentially related with RFI.

RNA sequencing (RNA-seq) has previously been used to detect gene expression associated with divergent RFI in cattle. Researchers have identified several RFI-related genes and pathways from cattle liver (Tizioto et al., 2017), skeletal muscle (Khansefid et al., 2017), blood (Xi et al., 2015), adipose tissue (Weber et al., 2016), and rumen epithelium (Kong et al., 2016). However, the reported differentially expressed genes (DEGs) differed significantly among studies, likely due to differences in breeds, age, sex, and tissue. For example, McKenna et al. (McKenna et al., 2021) found 11 DEGs, M. S. Salleh et al. (Salleh et al., 2017) found 70 (in Holsteins) and 19 (in Jerseys) DEGs, and Robert Mukiibi et al. (Mukiibi et al., 2018a) identified 72 (in Angus), 41 (in Charolais), 175 DEGs (in KC breed). Although some DEGs were obtained from these studies, none of the overlapped genes were commonly present in the above studies at the same time.

Nutrient digestion and absorption typically account for more than 10% of RFI variation (Herd and Bishop, 2000). The intestinal tract is the primary organ controlling these processes. The duodenum—the first digestive and absorptive organ of the intestinal tract—is vital for the absorption of glucose (Zhong et al., 2020), fat (Everard et al., 2019), vitamin B (Wang et al., 2019), calcium (Moine, 2018), zinc (Zhong et al., 2020), and iron (Andrews, 2008). Therefore, to better understand how the duodenum and its functions are associated with RFI phenotypes. The present study used RNA-seq technology and bioinformatics to identify the genes and functional pathways related to RFI in Chinese Qinchuan cattle. This study aimed to provide a broad perspective for understanding not only feed intake in farm animals.

## Materials and Methods

### Animals and RFI Calculation

Thirty healthy Qinchuan bulls of similar age (14–16 months) were selected from a bred population in Ningxia, China. Their initial body weight (BW_0_) was 280.6 ± 30.9 kg, and they were offspring of a sire bull. The animals were provided with the same diet for the duration of the experiment. During the trial period, cattle were fed in an independent room measuring 3 × 4 m. All animals had ad libitum access to water and feed and were weighed once per month.

The feed intake (FI) was measured daily from day 1 to day 81 using an automatic feeding system, and the ADFI was then calculated accordingly. The BW_0_ and final body weight (BW_81_) were recorded to calculate the average midpoint metabolic weight (MMBW^0.75^) and ADG. RFI was defined as the difference between the ADFI and EDFI using the following formula:

$$\mathrm{RFI}=\mathrm{ADFI}-\left(b_{0}+b_{1}\times \mathrm{ADG}+b_{2}\times {\mathrm{MMBW}}^{\text{0}\text{.}75}\right)$$

Here, the ADG and MMBW^0.75^ are the slope of the linear regression between BW and days of feeding and the midpoint metabolic BW0.75, respectively. The $b_{0}$ , $b_{1}$ and $b_{2}$ are the regression intercept, the partial regression coefficient of ADFI on ADG, and the partial regression coefficient of ADFI on MMBW^0.75^, respectively. Then, the cattle with positive and negative RFI values were categorized into the high- and low- RFI (HRFI and LRFI) groups, respectively. R language (version 3.6.1, https://www.r-project.org/) was used to perform relevant calculations, and *p* < 0.05 was set as the significance threshold. All results were expressed as mean ± standard deviation.

### RNA Isolation and Transcriptome Sequencing

After RFI calculation, the ten animals from the five highest HRFI and the five lowest LRFI groups were slaughtered after a 16-h fast following the guidelines of the Animal Ethics Committee of Ningxia University. The descending portion tissue of the duodenum (including the mucosa, submucosa, and muscularis externa layer) was collected within minutes after slaughter, washed with PBS solution, cut into pieces, and placed in sterile RNase- and DNase-free cryopreservation tubes, and then stored in liquid nitrogen. According to the manufacturer’s instructions from the TRIzol RNA extraction kit (Invitrogen, Carlsbad, CA, United States), approximately 500 mg of duodenal tissue sample was used for RNA extraction. The RNA quality of 10 samples was satisfied with RNA quality higher than 1.8 and RIN values higher than 7. The RNA sequencing libraries were constructed and sequenced by NovelBrain Biotechnology Co., Ltd. (Shanghai, China) using the Illumina HiSeq 4000 platform (Illumina, San Diego, California, United States), which sequencing length of the read was 150 bp with pair-end.

### Quality Control and Alignment

Base call data from the original binary base call files were converted to raw sequence data in FASTQ format. Subsequently, FastQC software (version 0.11.7, https://www.bioinformatics.babraham.ac.uk/projects/fastqc/) was used to evaluate sequencing quality. Briefly, reads containing standard adapters or poly-N sequences and low-quality reads were trimmed via Trim-galore software (version 0.6.6, https://www.bioinformatics.babraham.ac.uk/projects/trim_galore/). The clean reads with average base quality greater than 20 were selected for subsequent analyses. The HISAT2 software (version 2.2.1, http://daehwankimlab.github.io/hisat2/) was used to build the bovine genome index (including information on splicing sites, haplotypes, exons, and SNPs) and were aligned with the clean reads to the reference genome (BosTau9, https://hgdownload.soe.ucsc.edu/goldenPath/bosTau9/). The bovine reference genome and annotation files, downloaded from the UCSC database, were used for guiding transcript quantitative using the StringTie software (version 2.1.2, http://ccb.jhu.edu/software/stringtie/). Following this, gene expression was quantified using the transcript per million (TPM) value.

### Identification of Differentially Expressed Genes and Functional Annotation

After getting the TPM value, we perform a principal component analysis (PCA) to check the sample repeatability. As a result, six bulls - three of each group - were used for differential expressed analysis (Supplementary Figure.S1), where the LRFI group included SRR15183075, SRR15183066, SRR15183067, and HRFI group included SRR15183071, SRR15183073, SRR15183074. DEGs were detected using R package DESeq2 (version 1.24.0, http://www.bioconductor.org/packages/release/bioc/html/DESeq2.html). The fold change (FC) thresholds for identifying DEGs were Log2FC > 1 or Log2FC < −1, and a quality score greater than Q20, i.e., false discovery rate (FDR) less than 0.05.

To detect the biological functions of the DEGs, gene ontology (GO) and Kyoto Encyclopedia of Genes and Genomes (KEGG) pathway analyses were conducted online using DAVID (version 6.8, https://david.ncifcrf.gov/). In GO analysis, genes were grouped into the following domains: cellular composition, molecular function, and biological process. The Fisher test was used to calculate the significance level for each GO term and KEGG pathway, and the threshold for *p*-values was less than 0.05.

### Protein-Protein Interaction Network Construction and Selection of Crucial Modules

After obtaining the DEGs, they were mapped to the STRING database (version 11.0, https://string-db.org/) to acquire interaction information, with a confidence score over 0.9 used as the threshold. The Cytoscape application (version 3.6.1, https://cytoscape.org/) was utilized to construct complex interaction networks. The CytoHubba plugin in Cytoscape was used to detect hub genes through four centrality methods which were network topology analysis—Degree, edge percolated component (EPC), maximal clique centrality (MCC), and maximum neighborhood component (MNC), which are useful methods for identifying hub gene from PPI networks (Chin et al., 2014)— and the genes selected by all four methods were identified as the core gene set. The MCODE plugin was applied to identify key sub-networks and the seeds of nodes; these together formed the hub gene set (degree cutoff = 2, node score cutoff = 0.2, k-core = 2, and maximum depth = 100). Subsequently, genes from each module were subjected to GO and KEGG enrichment analyses.

### Weighted Gene Co-expression Network Analysis

WGCNA was applied to explore the relationship between gene expression and RFI, which was used to detect key genes, such as cancer (Andrews, 2008; Moine, 2018) and feed efficiency (Hou et al., 2018; Xu et al., 2020). WGCNA considers gene expression information and the related phenotype information and is thus more suitable for the data analysis of complex traits (Langfelder and Horvath, 2008; Mukiibi et al., 2018b; Higgins et al., 2019)-32]. In this work, the WGCNA study was conducted on ten bull’s transcriptome libraries. The co-expression networks of 10,161 genes, in which the average TPM of the LRFI group was greater than 6.96, were constructed using the R package WGCNA(version 1.69, https://horvath.genetics.ucla.edu/html/CoexpressionNetwork/Rpackages/WGCNA/). When the correlation coefficient was 0.8, the soft-thresholding power was 12 and the minimum number of genes in a module was set as 100. To combine potential parallel modules, 0.2 was selected as the threshold for cut height. To further understand the role of the expressed genes in the modules most closely related to RFI phenotypes, the DAVID Website was used for GO and KEGG analysis. *p* < 0.05 was set as the significance threshold. The R package ggplot2 tool (version 3.3.2, https://ggplot2.tidyverse.org/) was used to present the results. After interested modules, the function “exportNetworkToCytoscape” in R package WGCNA was used to calculate the interaction relationship among genes in interesting modules and selected the top 200 weighted edges for visualization.

### Validation of RNA-Seq via Quantitative Real-Time PCR

After differential analysis, a list of DEGs was obtained. To validate the accuracy of RNA-seq, qPCR tests were performed. Four pairs of special primers (MS4A1, PLN, KMO, and CHI3L4, which were selected by generating random numbers) were designed using the primer-blast tool (https://www.ncbi.nlm.nih.gov/tools/primer-blast) and synthesized by Sangon Biological Technology Co. Ltd. (Sangon, China) (The primer sequences information was presented in Supplementary Table S1). Based on manufacturer instructions, the first-strand cDNA was synthesized from six cattle’s total RNA (1 ug for each sample) using the One-Step gDNA Removal and cDNA Synthesis SuperMix (Vazyme, China). The CFX Real-Time PCR apparatus (Applied Biosystems, Warrington, United Kingdom) and SYBR Green Master Mix (Biomiga, San Diego, CA, United States) were used to perform qPCR analysis (Detailed procedures for qPCR reactions were attached in Supplementary Table S2). Each sample was examined in triplicate. The CT method was used to quantify differences in gene expression, and transcript levels were normalized based on GAPDH expression. The 2–ΔΔt method was employed to calculate relative expression.

## Results

### Performance and Feed Efficiency

According to the animal’s RFI value, the five highest and five lowest bulls were selected for differential analysis. All animal’s performance was presented in (Supplementary Table S3), and differential analysis for HRFI and LRFI group showed that the ADFI and RFI values were significantly higher in the HRFI group than in the LRFI group (*p* = 0.004 and *p* = 0.006, respectively) (Table 1). Based on ADFI, we found that the HRFI group consumed 11.97% more feed than the LRFI group, although ADG values did not show significant differences between the two groups. These results revealed that the LRFI group was able to more adequately improve feed efficiency and keep its ADG in line with the HRFI group.

**TABLE 1:** Analysis of differences for growth traits between the HRFI and LRFI groups (Mean ± Sd)


Traits	LRFI group^a^	HRFI group^b^	*p*-value^c^
BW0^d^, kg	285.75 ± 22.32	273.50 ± 20.28	0.448
BW81^e^, kg	419.25 ± 28.72	412.50 ± 35.10	0.776
MMBW0.75^f^, kg	80.51 ± 10.91	80.72 ± 26.69	0.076
ADFI^g^, kg	10.44 ± 0.23	11.86 ± 0.08	0.004
ADG^h^, kg/d	1.21 ± 0.047	1.187 ± 0.06	0.450
RFI^i^, kg	−0.84 ± 0.13	0.55 ± 0.044	0.006

^a^LRFI group: Containing the five lowest RFI values of five cattle.

b HRFI group: Containing the five highest RFI values of five cattle.

c *p*-value: level of a significance test, and its threshold were set at 0.05 and three decimal places retained.

^d^BW_0_: Initial body weight.

e BW_81_: Final body weight.

f MMBW^0.75^: Midpoint metabolic body weight.

g ADFI: Actual daily feed intake.

h ADG: Average daily gain.

i RFI: Residual feed intake.

### Gene Expression Profile

The numbers of raw and clean reads were more than 45 million and 41 million, respectively. After alignment to the BosTau9 genome, we obtained an average mapping rate of 86.19% for all samples (minimum = 83.3%, maximum = 88.4%). Therefore, our data quality satisfied the requirements for the subsequent analysis of genes differentially expressed between the HRFI and LRFI groups (Supplementary Table S4).

**FIGURE 1:**
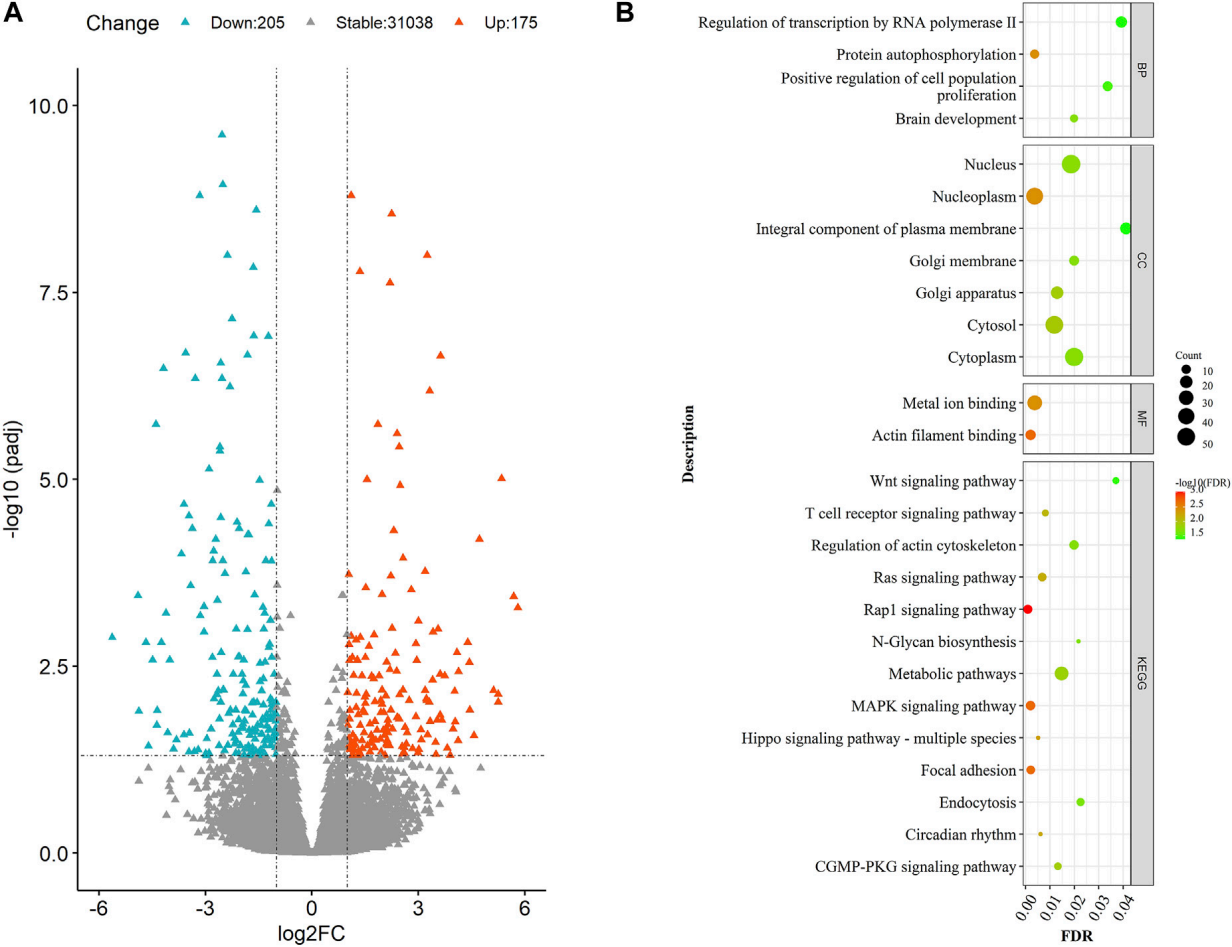
**(A)** Volcano diagram of DEGs. The volcano diagram exemplifies the size and significance of the genes expressed differentially between the HRFI and LRFI groups. The “Molokai blue” and “Luminous Orange” dots represent the down-regulated and up-regulated genes in the LRFI group, respectively. **(B)** GO and KEGG analysis of DEGs. The size and color of the dot represent the number of enriched genes and the magnitude of significance in **(B)**.

Among all the genes annotated in the bovine reference genome (Supplementary Table S5), a total of 380 were found to be DEGs in our study (Figure 1A). Of the 380 DEGs, 175 were up-regulated in the LRFI group, and 205 were down-regulated compared to the HRFI group. Among the up-regulated DEGs, 21 showed a fold change of over 16, and 79 showed fold changes ranging from 4 to 16. Meanwhile, among the down-regulated DEGs, 18 showed a fold change over 16, and 74 DEGs showed fold changes ranging from 4 to 16. The top 10 DEGs with the highest fold change (Table 2) were primarily involved in mitochondrial function or energy metabolism, such as NDUFS1, ATP2B4, and HADHB.

**TABLE 2:** Top ten upregulated and downregulated DEGs in LRFI group compared with HRFI group.


Symbol^a^	log2FC^b^	q-value^c^	Description^d^
CHD8	7.66	0.000	chromodomain helicase DNA binding protein 8
SSH2	6.18	0.000	slingshot protein phosphatase 2
PCBP2	5.80	0.001	poly (rC) binding protein 2
LOC407163	5.35	0.000	trappin 5
CERS5	5.25	0.007	ceramide synthase 5
TDP2	5.25	0.010	tyrosyl-DNA phosphodiesterase 2
WDR11	5.16	0.000	WD repeat domain 11
STYX	5.11	0.007	serine/threonine/tyrosine interacting protein
H2AFY	4.72	0.000	H2A histone family, member Y
NDUFS1	4.57	0.027	NADH: ubiquinone oxidoreductase core subunit S1
TADA2A	−4.40	0.000	transcriptional adaptor 2A
VPS37C	−4.50	0.003	VPS37C, ESCRT-I subunit
ATP2B4	−4.61	0.037	ATPase plasma membrane Ca2+ transporting 4
SLC6A20	−4.69	0.002	solute carrier family 6 member 20
LRRC28	−4.88	0.013	leucine rich repeat containing 28
WAC	−4.89	0.000	WW domain-containing adaptor with coiled-coil
CLOCK	−4.90	0.000	clock circadian regulator
TXLNA	−5.63	0.001	taxilin alpha
SMTN	−6.27	0.000	smoothelin
BAZ2A	−6.37	0.003	bromodomain adjacent to zinc finger domain 2A

a Symbol = gene symbol in NCBI.

b Log2FC = Logarithm of fold change with a base of 2, the value was rounded to two decimal places.

c q-value = Significance test probability value, the value was rounded to three decimal places.

d Description = Description of genes.

### Functional Enrichment of Differentially Expressed Genes

To explain the function of 380 DEGs, we had them performed a functional enrichment analysis. Results have shown that most DEGs were enriched in processes directly relevant to material metabolism (Figure 1B, Supplementary Table S6 and Supplementary Table S7), including “N−Glycan biosynthesis,” “Metabolic pathways,” which showed enrichment for the highest number of genes in the study; “Endocytosis pathway,” which were involved in nutrient absorption in intestinal epithelial cells; and “Circadian rhythm,” which regulated animal behavior. Meanwhile, we also found gene enrichment in the “WNT,” “MAPK,” and “T-cell receptor” signaling pathways, which were involved in the regulation of the immune response and nutrient metabolism. The enrichment analysis of 380 DEGs revealed that these genes were involved in functions that tend to be related to energy metabolism, material metabolism, and the regulation of animal behavior.

### Identification of Critical Genes and Pathways via Protein-Protein Interaction Network Analysis of DEGs

**FIGURE 2:**
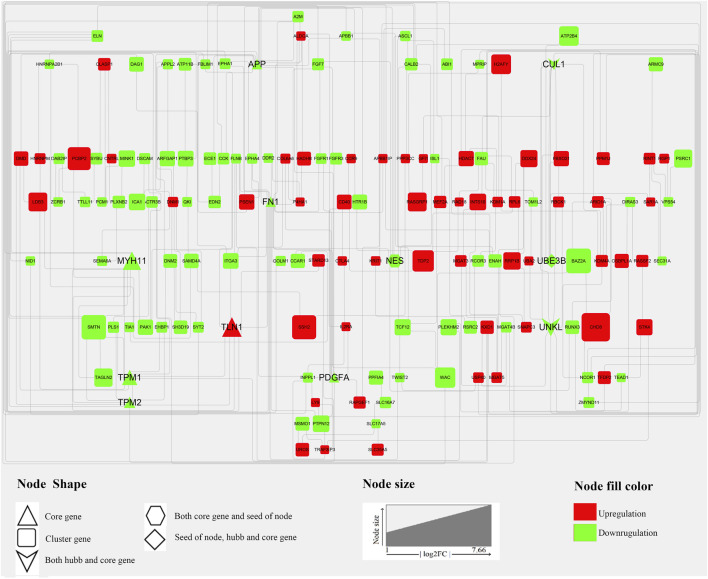
PPI network of DEGs. In total, 235 nodes and 383 interaction associations were detected. The red and green nodes represent the up-and down-regulated genes, respectively. The shapes “Triangles,” “hexagon,” “Square,” “diamond” and “V” denote the “Core gene,” “Both core gene and seed of node,” “Cluster gene,” “seed of node, hub, and core gene,” and “Both hub and core gene.” The “core genes” represent the overlap of the top 20 genes obtained using Degree, DMNC, MCC, and MNC. “hub genes” represent those genes which calculated by analyzing tools in Cytoscape from the PPI network, and the “seed of node” are those at the seed nodes of modules 1 or 2. “cluster genes” represent members of the network.

**FIGURE 3:**
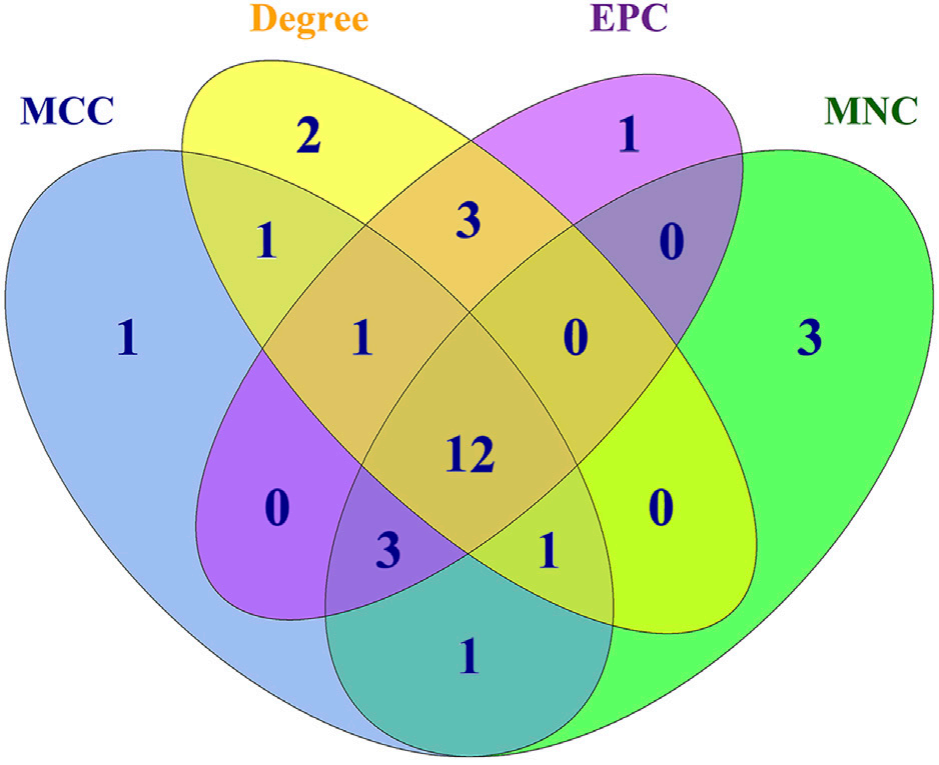
Overlap of genes identified using the four methods using Cyto-Hubba plugin in PPI. Four methods—Degree, DMNC, MCC, and MNC—were applied to identify significant hub genes using the four centrality methods. Different colors denote divergent algorithms. The intersections indicate the common DEGs. The elements common to all four methods were identified as the 12 core genes: FN1, TPM1, TPM2, UNKL, MYH11, RNF4, CUL1, UBE3B, APP, PDGFA, TLN1, and NES.

**FIGURE 4:**
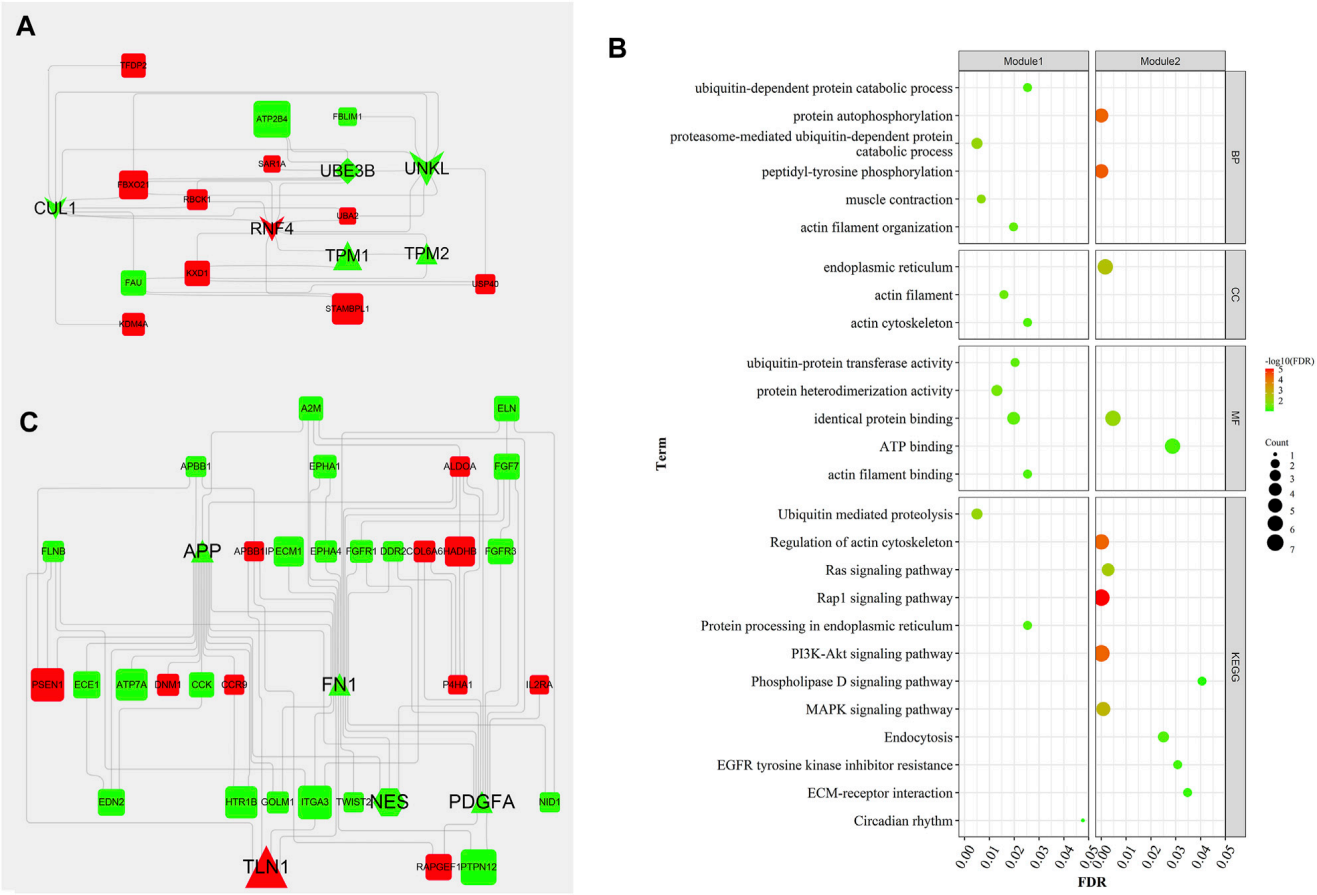
Two hub modules from the PPI network and their enrichment analysis. Module 1 (MCODE score = 4.353), including 18 nodes and 37 edges **(A)**, and module 2 (MCODE score = 3.771), containing 36 nodes and 66 edges (**C**), were constructed from the PPI network of the DEGs using MCODE. The color and shape represent the same values as described in Figure 2. The seed nodes in modules 1 and 2 were RNF4 and FN1, respectively. **(B)** represent the bubble diagrams for gene enrichment in modules 1 and 2, respectively. The color and size of the dot represent the number of enriched genes and the magnitude of significance, respectively.

Generally, genes show interaction networks and a complex trait controlled by micro-effective polygene. In the PPI network analysis for the identified DEGs, we constructed a gene interaction network that contained 235 nodes and 383 edges (Figure 2). Based on this network, twelve core genes were screened using the CytoHubba plugin in Cytoscape software, which was the overlap of four methods ranked top 20 by its value—Degree, EPC, MCC, and MNC. These genes were FN1, TPM1, TPM2, UNKL, MYH11, RNF4, CUL1, UBE3B, APP, PDGFA, TLN1, and NES (Figure 3). Most core genes were involved in important biological regulatory functions related to mitochondrial function and energy metabolism. For example, CUL1 was reported to play an essential role in oxidative stress, mitochondrial stress, and basal respiration. Meanwhile, TPM1 and TPM2 were shown to be involved in mitochondrial pathways and protein synthesis. Additionally, two significant modules (module 1, MCODE score = 4.53, Figure 4A; module 2, MCODE score = 3.71, Figure 4C) were constructed based on the PPI network of the DEGs, and the seed nodes were RNF4 and FN1, respectively. Module 1, which contained 18 nodes and 37 edges, was enriched for GO terms such as “identical protein binding,” “metal ion binding,” and “muscle contraction” and for KEGG pathways “ubiquitin-mediated proteolysis,” “adrenergic signaling in cardiomyocytes,” and “protein processing in endoplasmic reticulum” (Figure 4B). Module 2, which contained 36 nodes and 66 edges, was enriched for GO terms such as “protein autophosphorylation,” “peptidyl-tyrosine phosphorylation,” and “ATP binding” and for KEGG pathways such as “PI3K-Akt signaling pathway,” “MAPK signaling pathway,” and “Rap 1 signaling pathway” (Figure 4B). Through module analysis, several vital pathways were identified. Notable among these were the PI3K-Akt and MAPK signaling pathways, involved in inflammation and metabolism, and the “muscle contraction” pathway involved in energy metabolism.

### Weighted Gene Co-expression Network Analysis

**FIGURE 5:**
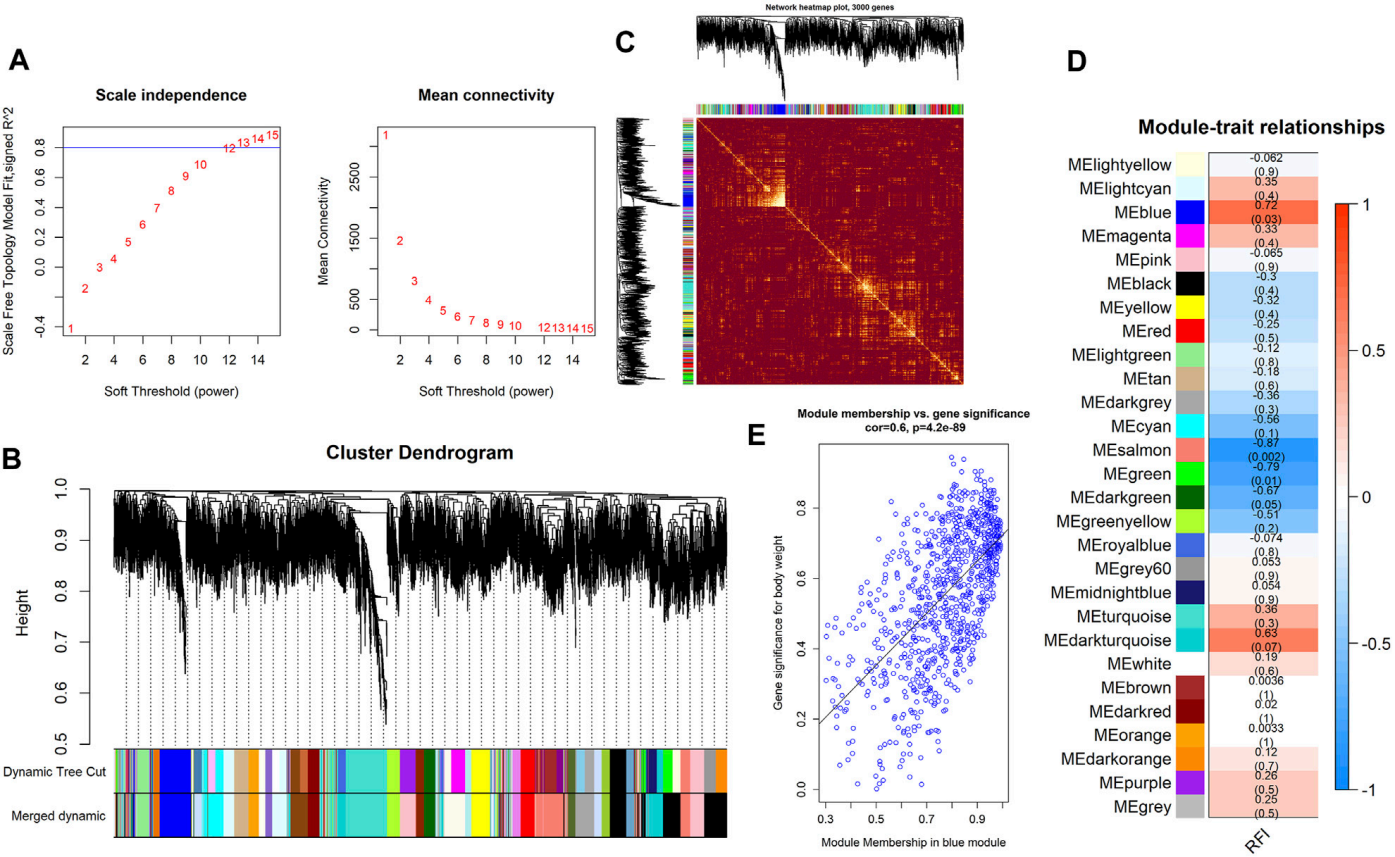
Key findings from the module–trait correlations analyses. **(A)** The analysis of the scale-free fit index **(left)** and mean connectivity for diverse soft-thresholding powers **(right)**. **(B)** Clustering dendrogram of DEGs associated with RFI. **(C)** Network heatmap in the co-expression modules (the yellow color scale indicates the degree of overlap between functional modules). **(D)** Heat map of the correlations between modules and RFI (each cell contains the correlation coefficient and its *p*-value). **(E)** Significance of genes contributes to RFI in the blue module (one dot denotes a gene in the blue module).

**FIGURE 6:**
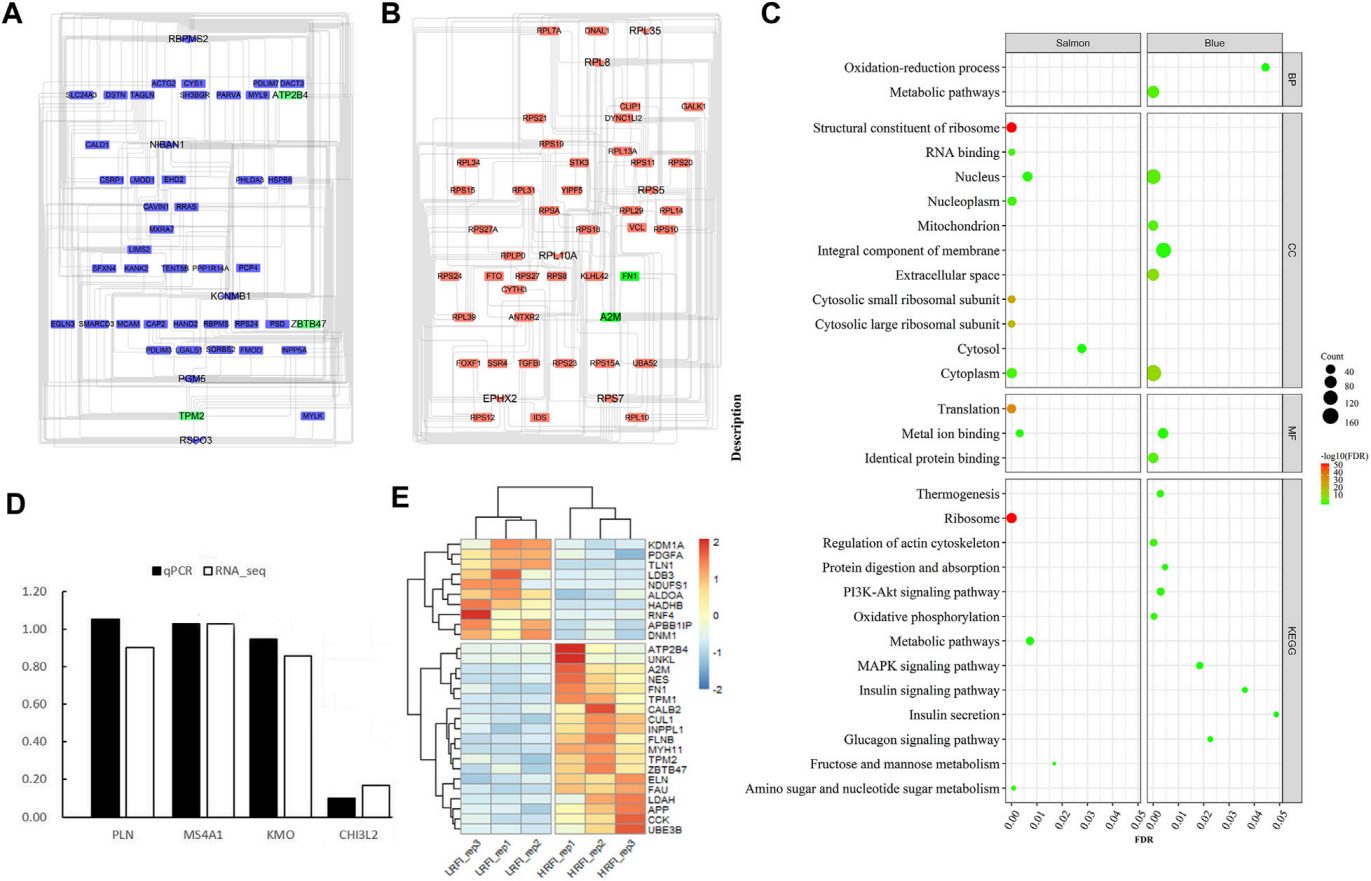
PPI and enrichment analysis of DEGs in the salmon and blue modules (considering the number of interaction networks, the top 200 genes in the weighted interaction network were selected to construct the interaction network). **(A)** Interaction relationship in the blue module, consisting of 47 nodes and 200 edges. **(B)** Interaction relationship in the salmon module, composed of 48 nodes and 197 edges. In the blue and salmon modules, the top 40 genes were defined as hub genes. The shape “Ellipse” represented the top 20 genes, and the shape “hexagon” denoted the genes ranking 21–40. **(C)** represent the enrichment analysis of genes in the blue and salmon modules. In **(C)**, the color and size of the dot represent the number of enriched genes and the magnitude of significance, respectively. **(D)** qPCR results for four randomly selected genes. **(E)** Heatmap of all candidate genes identified. Blue to yellow fading means gene expression increases, positive and negative numbers do not indicate positive or negative expression but up-or down-regulation.

Using WGCNA, 27 co-expression modules were constructed. The turquoise module had the highest number of genes (1743 genes), followed by the blue (900 genes), brown (806 genes), yellow (595 genes), and salmon modules (269 genes) (Figures 5A,B). These modules were independent of each other (Figure 5C). Analysis for module–trait correlations indicated that various modules were associated with RFI, with the salmon module showing the highest correlation (*r* = −0.87, *p* = 0.002), followed by the blue module (*r* = 0.72, *p* = 0.03). It suggested that the genes in salmon and blue modules may be closely associated with RFI (Figure 5D). Noteworthy, several genes in the blue module such as DDR2, ZDHHC2, MAP3K20, SYNM, RAB23 had high gene significance for RFI, and there was a strong correlation between them (module membership >0.83) (Figure 5E). In the salmon module, genes−for example, KLHL42, TMEM251, ABI3BP, NKX2-3, FOXF1− had high gene significance for RFI, and its module membership was at least −0.79 (Supplementary Table S2). After obtaining the salmon and blue module, we constructed the PPI networks for the top 200 weighted edges of the blue and salmon modules, and they consisted of 47 nodes and 200 edges, and 48 nodes and 197 edges, respectively (Figures 6A,B). Enrichment analysis of genes in the blue module shown that they were mainly enriched in energy-related metabolism such as “Oxidative phosphorylation,” “Mitochondrion,” “MAPK signaling pathways,” and “PI3K-Akt signaling pathways,” and substance-related metabolism such as “Glucagon signaling pathway” and “Metabolic pathways” (Figure 6C). In the salmon module, genes were mainly enriched in substance-related metabolism such as “metabolic pathways,” “amino sugar and nucleotide sugar metabolism,” “amino sugar and nucleotide sugar metabolism,” and “fructose and mannose metabolism” (Figure 6C). In terms of enrichment analysis, both blue and salmon modules were involved in the metabolism of substances or energy, suggesting that the metabolism process may be closely related to RFI.

To identify the hub genes in the two modules, we obtained the intersection of the top 200 genes ranked based on weighted interaction and the DEGs that were found to be critical core genes. The following genes were identified: FN1, TPM2, A2M, ZBTB47, and ATP2B4. Among these, they take part in many biological processes (Supplementary Figure S3). The FN1 gene is known to be involved in the “WNT,” “MAPK,” “PI3K-Akt,” and “TGF-β” pathways, which are closely related to host immune processes.

### Validation of RNA-Seq Results

To validate the accuracy of the RNA-seq profiles, we selected randomly four expressed genes for qPCR, including PLN (phospholamban), MS4A1 (membrane-spanning 4-domains A1), KMO (kynurenine 3-monooxygenase), and CHI3L2 (chitinase 3-like 2). The sample used for qPCR was the same as that used for RNA-seq. The qPCR results have shown that the level of gene expression trend was similar to that observed in RNA-seq, indicating that the RNA-seq results were accurate and reliable (Figure 6D).

## Discussion

Improving feed efficiency in beef cattle is a long-term and far-reaching breeding goal in livestock production. Over the last few years, various tissues from beef cattle with different RFI levels have been examined using transcriptome sequencing to identify marker genes or related biological processes associated with feed efficiency. These tissues are liver (Hui-Zeng, 2018; Wang, 2020), rumen (Kong et al., 2016), muscle (Yang et al., 2020), and fat tissue (Grubbs et al., 2013), as well as blood (Khansefid et al., 2017). It is clear that the DEGs and pathways associated with RFI screened based on different tissue samples are both distinct and linked. As an important organ in the digestive tract, the duodenum is closely related to digestion and nutrient absorption, such as glucose (Zhong et al., 2020) and fat (Everard et al., 2019); Here, we performed transcriptome sequencing of duodenal tissue from Qinchuan cattle, following GO classification, KEGG pathway enrichment, PPI network construction, and WGCNA analysis. We tried to investigate key DEGs and pathways associated with RFI from duodenal tissue and identify potential regulation processes that could help improve cattle feed efficiency in the future.

As expected, we found gene expression differences between cattle with different RFI levels in duodenal. Studies show that feed efficiency is closely related to energy metabolism. Individuals with high feed efficiency can synthesize ATP more efficiently and produce less reactive oxygen species (ROS) via enhanced mitochondrial function (Sierant, 2019). Low ROS production prevents oxidative damage to lipids and proteins while allowing for reduced mitophagy and protein turnover (Agarwal et al., 2019). Moreover, animals with high feed efficiency have greater resistance to oxidative stress, resulting in a lower inflammatory response and less impaired growth, which results in more energy being used for their growth and development (Dias et al., 2019). In our study, we detected 29 key genes (Figure 6E) related to RFI. Of those genes, three were closely associated with mitochondrial energy metabolism (NDUFS1, HADHB, and ALDOA) and were significantly upregulated in the LRFI group. NDUFS1 encodes one subunit of mitochondrial complex I, which is mainly involved in transferring electrons and maintaining the redox balance (Zou et al., 2021) in the first step of oxidative phosphorylation (Bertile et al., 2021). Knockdown of the NDUFS1 gene reduces membrane potential and mitochondrial mass in cardiomyocytes while also causing increased ROS production (Ni et al., 2019). It has also been shown that NDUFS1 expression is downregulated in the brains of food-restricted mice, suggesting that NDUFS1 downregulation makes mice more responsive to stress and increases ROS (Gielisch and Meierhofer, 2014). Mutations in NDUFS1 can affect the gene product and destabilize the N module of complex I, which interrupted electron tunneling between N4 and N5 subunits, causing an increase in the NADH/NAD + ratio in the electron respiratory chain. This results in a decrease in the amount of NAD+ (Esterhuizen et al., 2017), leading to a blockage in the conversion of malate to oxaloacetate in the tricarboxylic acid cycle (TCA), which causes TCA inhibition via negative feedback due to product accumulation (Schütt et al., 2012; Zeng et al., 2016).

ALDOA encodes a glycolytic enzyme that increases glucose utilization, contributes to aerobic glycolysis (Fu et al., 2018), and affects oxidative stress (Gaiying et al., 2021). ALDOA knockdown causes a decrease in the concentration of ATP (Li and Schulz, 1988), whereas its overexpression inhibits oxidative stress in cardiomyocytes under both hypoxic and normal conditions via the VEGF/Notch/Jagged 1 pathway (Pietrocola et al., 2015). Another key DEG is HADHB, which plays a critical role in the β-oxidation of fatty-acyl CoA as the key enzyme catalyzing the final and rate-limiting step in long-chain fatty acid oxidation (Kao et al., 2006). Mutations in the HADHB gene result in a deficiency in the mitochondrial trifunctional protein, which prevents the body from metabolizing long-chain fatty acids and further leads to a deficiency of acetyl coenzyme A, thereby inhibiting the TCA (Shao et al., 2019). Increased expression of the HADHB gene promotes fatty acid oxidation and reduces the accumulation of fat in the liver (Iakova et al., 2020; K2-F4 interplay coor, 2017). The growth and development of an organism require large amounts of energy, primarily derived from the TCA cycle. NADH is produced mainly through the electron respiratory chain to generate large amounts of ATP for life activities. The three identified DEGs–HADHB, ALDOA, and NDUFS1–are essential for fatty acid β-oxidation (Kao et al., 2006), glycolysis (Fu et al., 2018), and the function of the electron transport chain complex I, respectively (Zou et al., 2021). Moreover, other DEGs, such as CUL1 signigicantly down-regulated in the LRFI group (knockdown can lead to oxidative injury) (Goo et al., 2017), RFN4 signigicantly up-regulated in the LRFI group (deficiency of it can result in mitochondrial stress) (Syota et al., 2008), LDAH signigicantly down-regulated in the LRFI group (it plays a primarily lipogenic role (MoranKatz et al., 1998)), INPPL1 signigicantly down-regulated in LRFI group (it shows a capacity to impair insulin signaling (Ueno and Nakazato, 2016)), were also involved in substance or energy metabolism. In general, those genes regulate directly or indirectly mitochondrial function, reduce ROS production, and increase stress resistance, which may contribute to higher feed efficiency.

In addition to energy metabolism, the regulation of feed intake behavior could also lead to higher feed efficiency. CCK mainly regulates feeding behavior by stimulating the vagal nerve through the CCK receptor (CCKAR) (Cookand, 2004) and then terminates feeding behavior while promoting metabolism (Pekas and Trout, 1990). Oral treatment with anti-CCK antibodies improves feed efficiency and the growth rate in broilers (Cawthon and Serre, 2021), and immunization of piglets with CCK can dramatically increase feed intake and growth rate (Gamble et al., 2013). One feature of diet-induced obesity (DIO) in humans has reduced sensitivity to CCK, and vagal afferent neurons phenotypic flexibility is lost in DIO (Mei and Zhu, 2015). Moreover, CCK also inhibits gastric emptying and feed intake (Herd and Arthur, 2009). In the present study, the expression of CCK was signigicantly down-regulated in the LRFI group compare to control, suggesting the critical role of CCK in beef cattle. Therefore, we speculate that animals with high RFI eat more owing to reduced sensitivity to CCK and impaired sensitivity of vagal afferents, resulting in a delayed satiety signal, which may ultimately lead to an increase in feed intake. However, this hypothesis needs to be validated by further functional experiments.

It is well-known that a large number of genes generally controls trait, with different genes having different degrees of effects. Many genes that regulate a particular life process are often involved in specific signaling pathways that work together to control that life process through a cascade of signal transduction reactions (Liu et al., 2016). Research has demonstrated that metabolic processes and factors—protein turnover, tissue metabolism, body composition, and physical activity—can explain 73% of RFI variation (Jin et al., 2019). Generally, RFI is thought to primarily be associated with metabolic processes (Horodyska et al., 2017) such as fatty acid oxidation (Casal et al., 2020), protein synthesis (Kenny et al., 2018), and energy metabolism (Wu et al., 2020), and with appetite regulation (Zhao et al., 2020). In our study, the MAPK and PI3K-Akt signaling pathways were two of the most commonly observed pathways. Studies have demonstrated that the MAPK signaling pathway is associated with lipid (Wang et al., 2018; Zhang et al., 2018) and energy metabolism (Zheng et al., 2016; Kim et al., 2018) that regulates glucose metabolism (Long et al., 2015). Moreover, studies have also shown that PI3K-Akt signaling is involved in glucose (Ruzzi et al., 2020), lipid (Fu et al., 2020), and energy metabolism [79, 80]. Based on the present results of the enrichment analysis, it appears that the identified DEGs may regulate feed efficiency through signaling pathways related to energy metabolism and substance metabolism. Our functional annotation of key genes revealed that they are indeed involved in regulating energy metabolism, lipid metabolism, oxidative stress, and feeding behavior. Furthermore, similar results were obtained from enrichment analysis, PPI network analysis, and WGCNA analysis.

Although we may have identified key genes and signaling pathways associated with RFI in the duodenum, this study still has a few limitations. First, the sample size used for RNA-seq and WGCNA needs to be expanded, and our findings should be validated with other datasets, such as data from RNA-seq analyses of liver or other tissues of the tested individual, or integrated with other genomic data, such as QTLs, to broaden our understanding of molecular regulation and animal phenotype.

## Conclusion

Duodenal DEG and their signaling pathway may be more likely to improve feed efficiency by strengthening mitochondrial function for energy efficiency. The expression upregulation of genes related to energy metabolism and feeding behavior may play an essential role in regulating feed efficiency in livestock. However, this result still requires further functional experiments to verify it.

## Data Availability

The datasets generated for this study can be found in the [ENA database] [https://www.ebi.ac.uk/ena/browser/view/PRJNA747740?show=reads], and the accession number is PRJNA747740.
