# Supplementary figures and images for: Characterization and Duodenal Transcriptome Analysis of Chinese Beef Cattle With Divergent Feed Efficiency Using RNA-Seq
Source: Front Genet. 2021 Oct 5;12:741878. doi: 10.3389/fgene.2021.741878 (PMC8524388; doi:10.3389/fgene.2021.741878)

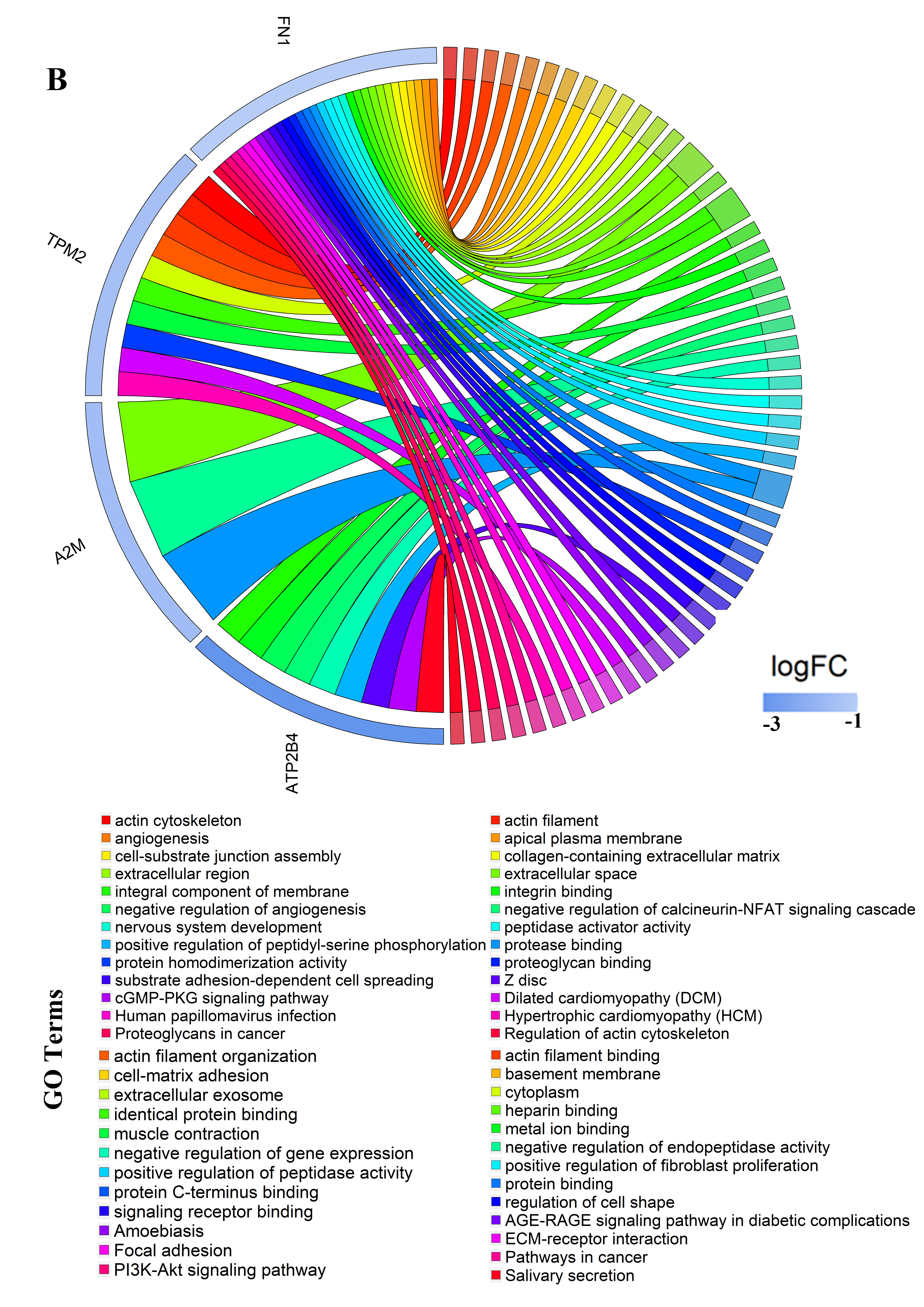

Supplement: Supplementary file 2 [file Image3.JPEG]

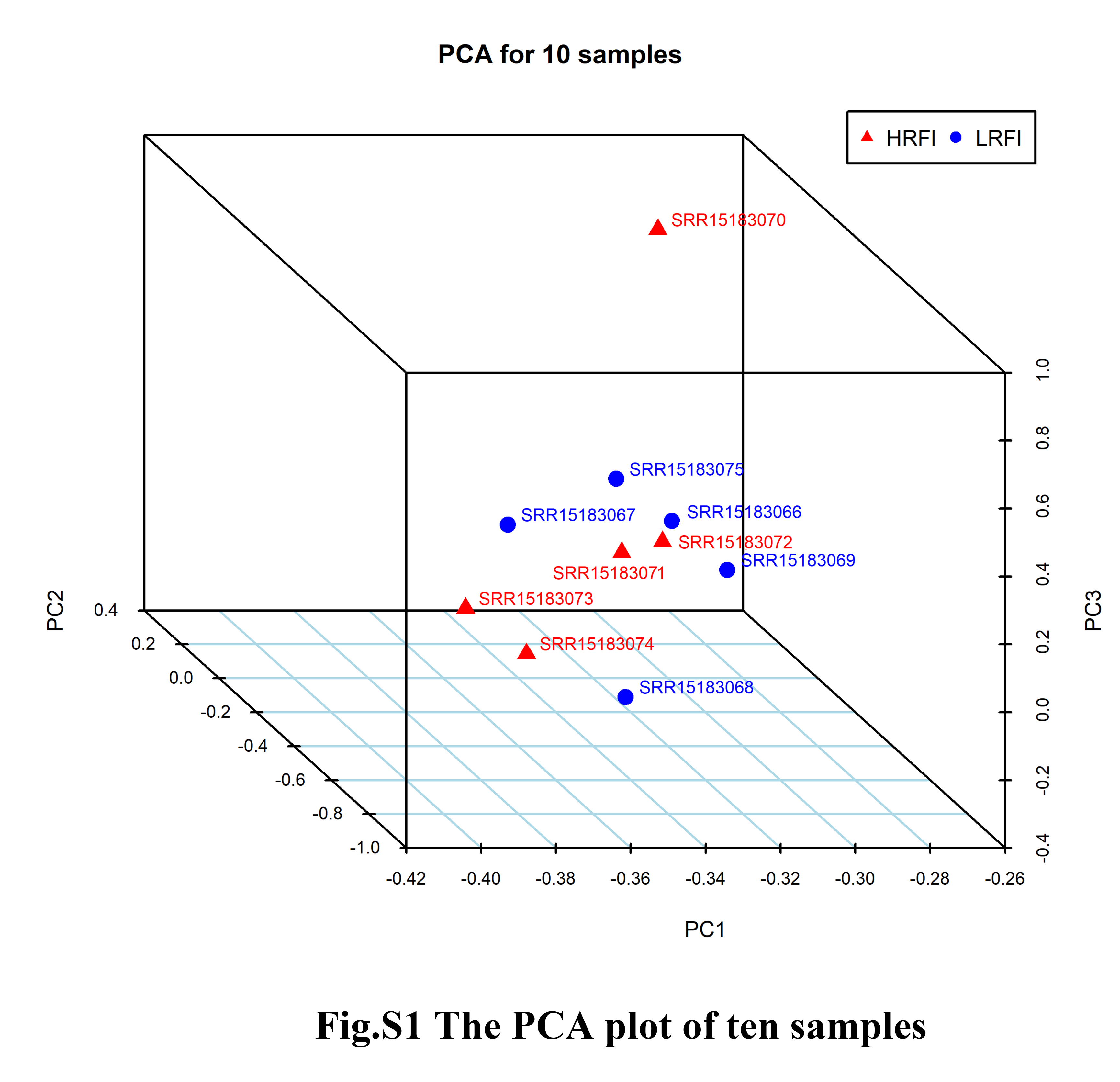

Supplement: Supplementary file 5 [file Image1.JPEG]

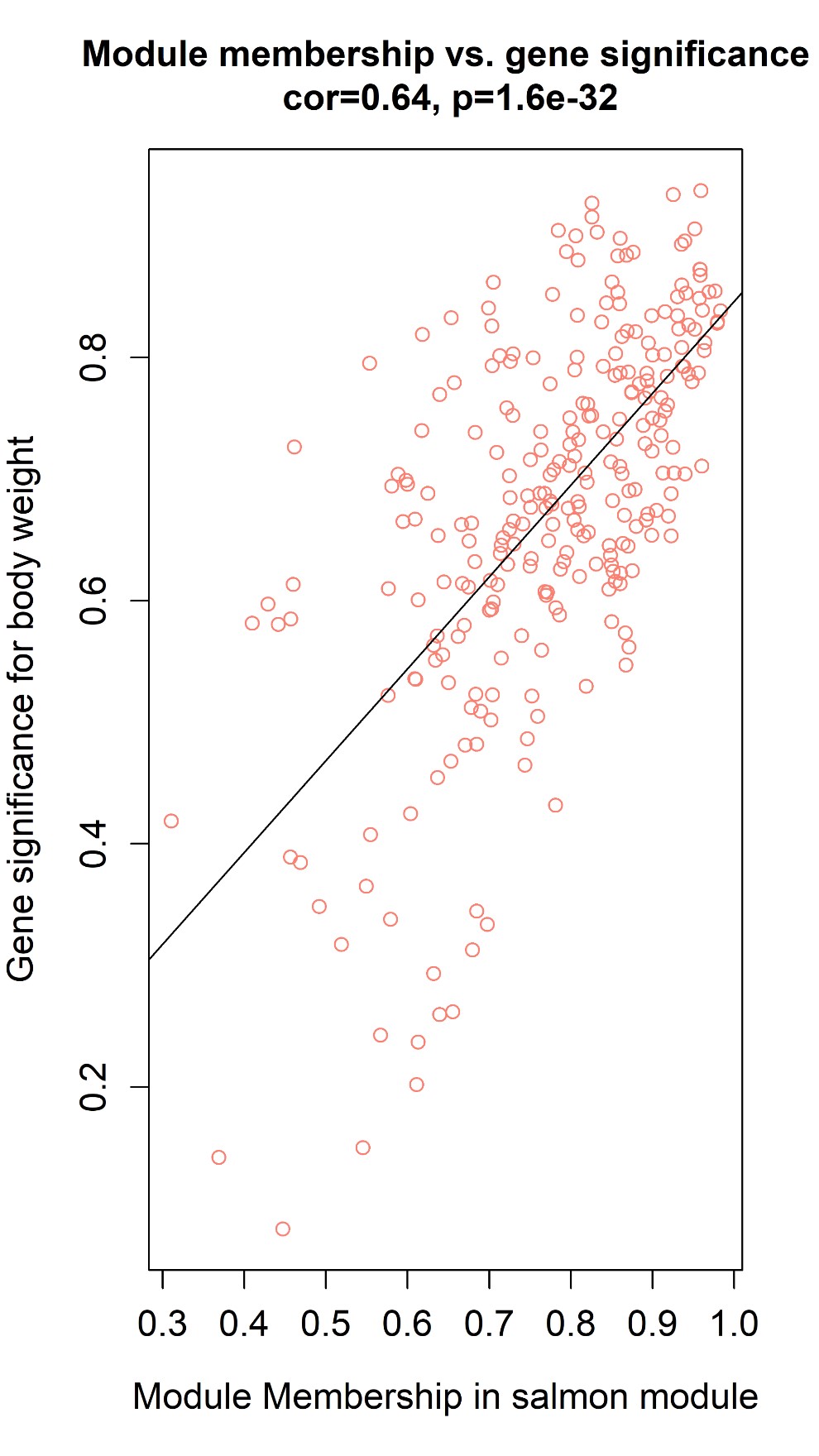

Supplement: Supplementary file 6 [file Image2.JPEG]
