# Supplementary material for: Characterization and Duodenal Transcriptome Analysis of Chinese Beef Cattle With Divergent Feed Efficiency Using RNA-Seq
Source: Front Genet. 2021 Oct 5;12:741878. doi: 10.3389/fgene.2021.741878 (PMC8524388; doi:10.3389/fgene.2021.741878)
Supplement: Supplementary file 4 [file Table8.DOCX]

**Supplementary 10:** Model availability

**Model availability for calculating RFI**: Calculation formula referred to an article by Koch(Koch, Swiger et al. 1963). With this model, we arrive at the following equation:

$$RFI=FI-(9.252-0.867\times ADG+0.038\times{MMBW}^{0.75})$$

And the model reasonableness and parameter check were indicated in the following tables, respectively.

| Model Reasonableness Check | | | | | |
| --- | --- | --- | --- | --- | --- |
|  | df | SS | MS | F | Significance F |
| Regression analysis | 2 | 1.526 | 0.763 | 3.062 | 0.064 |
| Residuals | 26 | 6.48 | 0.249 |  |  |
| Total | 28 | 8.006 |  |  |  |

| Model Parameter Check | | | | |
| --- | --- | --- | --- | --- |
|  | Coefficients | Standard error | t Stat | P-value |
| Intercept | 9.252 | 1.287 | 7.187 | 0.000 |
| ADG | -0.867 | 0.39 | -2.223 | 0.035 |
| MMBW0.75 | 0.038 | 0.018 | 2.094 | 0.046 |

**Sequencing data quality control**: trim_galore software (version=0.6.6) was used to perform quality control for raw data and its code as followings:

trim_galore --paired --retain_unpaired --quality 25 --length 36 –stringency 3 --cores 48 -o output_dir read1 read_2

**Index construction**: Hisat2 software (version=2.2.2) was used to construct index and its code as followings:

hisat2-build -p 48 bosTau9.fa --snp snp_info.snp --haplotype snp_info.haplotype –exon AnnotationBosTau9.exon --ss AnnotationBosTau9.ss out_dir

**Mapping**: Hisat2 software (version=2.2.2) was used to map reads to reference genome and its code as followings:

hisat2 -p 48 -x hisat2_index -1 read_1 -2 read_2 -S output_dir

**Quantification of gene expression**: StringTie software (version=2.1.2) was used to quantify the level of gene expression and its code as followings:

stringtie -e -B -p 48 -G Annotaotion.gtf -o filename -A filename filename.bam
